# *VPB1* Encoding BELL-like Homeodomain Protein Is Involved in Rice Panicle Architecture

**DOI:** 10.3390/ijms22157909

**Published:** 2021-07-24

**Authors:** Mu Li, Debao Fu, Tingting Xu, Changyin Wu

**Affiliations:** National Key Laboratory of Crop Genetic Improvement, Huazhong Agricultural University, Wuhan 430070, China; debaofu@webmail.hzau.edu.cn (D.F.); xtt8831@webmail.hzau.edu.cn (T.X.)

**Keywords:** inflorescence architecture, BLH homedomain protein, branching pattern, verticillate primary branch, transcriptome analysis, hormone pathways

## Abstract

Inflorescence architecture in rice (*Oryza sativa*) is mainly determined by spikelets and the branch arrangement. Primary branches initiate from inflorescence meristem in a spiral phyllotaxic manner, and further develop into the panicle branches. The branching patterns contribute largely to rice production. In this study, we characterized a rice *verticillate primary branch 1*(*vpb1*) mutant, which exhibited a clustered primary branches phenotype. Gene isolation revealed that *VPB1* was a allele of *RI*, that it encoded a BELL-like homeodomain (BLH) protein. *VPB1* gene preferentially expressed in the inflorescence and branch meristems. The arrangement of primary branch meristems was disturbed in the *vpb1* mutant. Transcriptome analysis further revealed that *VPB1* affected the expression of some genes involved in inflorescence meristem identity and hormone signaling pathways. In addition, the differentially expressed gene (DEG) promoter analysis showed that *OsBOPs* involved in boundary organ initiation were potential target genes of VPB1 protein. Electrophoretic mobility shift assay (EMSA) and dual-luciferase reporter system further verified that VPB1 protein bound to the promoter of *OsBOP1* gene. Overall, our findings demonstrate that *VPB1* controls inflorescence architecture by regulating the expression of genes involved in meristem maintenance and hormone pathways and by interacting with *OsBOP* genes.

## 1. Introduction

Inflorescence is the clusters of flowers arranged on a stem, and it comprises a main branch and lateral branches with a complicated arrangement [1,2,3]. The inflorescence architecture of higher plants contributes not only to plant morphology but also to plant reproduction, and further affecting the final grain yield in crops [4]. The panicle-type inflorescences are characteristics of grasses such as maize (*Zea mays*) and rice (*Oryza sativa*) [5]. Maize has two types of inflorescences, male tassel and female ear, which are different in morphology and branching pattern [6]. Rice inflorescence, also known as ‘panicle’, during panicle development, and shoot apical meristem (SAM) is transformed into the inflorescence meristem (IM) after transition from vegetative phase to reproductive phase, IM successively generates the primary and secondary branch meristem (PBM and SBM), floret meristem (FM), and spikelet meristem [7]. The main stem of rice panicle has primary and secondary branches, which are arranged in a spiral phyllotaxy [8]. Thus, the panicle branching patterns determine rice panicle architecture and eventually affect grain yield in rice [9]. 

So far, a large number of genes involved in regulating inflorescence architecture in rice have been identified, such as *LAX PANICLE1* (*LAX1*) and *LAX2* participating in the formation of axillary meristem (AM) in rice [10,11] and *ABERRANT PANICLE ORGANIZATION 1* (*APO1*) positively regulating the number of spikelets and primary branches and affecting the attributes of floral organs and the identity of flowers [12]. *APO2* has been reported to regulate the transition from rice vegetative growth to reproductive growth and to control the development of panicle branches, and it can directly interact with *APO1* to control the inflorescence and flower development [13]. The functional loss of either *FLORAL ORGAN NUMBER1* (*FON1*) or *FON2* causes the enlargement of the floral meristem, thus resulting in the increased floral organs [14,15]. *ABERRANT SPIKELET AND PANICLE1* (*ASP1*; also known as *OsREL2*) regulates different aspects of rice development and physiological responses, such as the development of panicles, branches, and spikelets [16,17]. *FON2* and *ASP1* are involved in the negative regulation of stem cell proliferation in both inflorescence meristems and flowers [18]. *TILLERS ABSENT1* (*TAB1*) plays an important role in initiating the rice axillary meristems, but this gene is not involved in maintaining the established meristem [19]. *TAW1* regulates inflorescence development by enhancing the activity of inflorescence meristems to inhibit the transformation from inflorescence meristems to spikelet meristems [20]. Those above-mentioned genes mainly control the length and the number of branches and meristem maintenance. However, our knowledge of the genetic mechanisms underlying branching patterns including branch phyllotaxy and internode elongation in rice remains limited.

Interestingly, the three-amino-acid-loop-extension (TALE) class of homeoproteins falls into two subfamilies, *KNOTTED1-like* homeobox (*KNOX*) and *BELL1-like* homeobox (*BLH*), which have been reported to control meristem formation and maintenance, organ position in plant, and organ morphogenesis [21]. For example, in *Arabidopsis thaliana*, two paralogous *BLH* genes, *PENNYWISE* (*PNY*) (also known as *BELLRINGER* (*BLR*), *REPLUMLESS* (*RPL*), or *VAAMANA* (*VAN*)) and POUND-FOOLISH (*PNF*), play significant roles in maintaining the SAM and the development of the inflorescence architecture [22,23,24,25,26,27,28,29]. Loss-of-function *PNY* gene causes the altered phyllotaxy, including irregular internode elongation, clusters of branches and flowers on the stem, and eventually reducing apical dominance [30]. Furthermore, *PNY* is involved in the establishment of normal phyllotaxis by repressing the expression of *PME5* (pectin methylesterase) in the meristem and the maintenance of phyllotaxis by activating *PME5* in the internode [31]. BLH proteins can interact with KNOX proteins to form heterodimer. For example, PNY interacts with the SHOOTMERISTEMLESS (STM) and BREVIPEDICELLUS (BP). The double mutant *bp/pny* exhibits synergistic phenotype of the short internodes interspersed with the long internodes and the increased branches [30]. The interaction between PNY and STM maintains the boundary between floral primordia and inflorescence meristem, and the SAM function in *Arabidopsis* requires both PNY and STM [32,33]. In addition, ChIP-seq results reveal that *PNY* interacts with many of the key genes regulating stem morphogenesis and controling the oriented growth by directly repressing organ boundary genes [34]. In maize, the two *BLH* genes, *BLH12* and *BLH14*, are close homologs of *PNY* and *PNF*, and double mutant *blh12/blh14* causes abnormality in internode pattern and vascular bundles anastomosis as well as indeterminate branch formation in the tassel [35]. 

In rice, one *BLH* gene *qSH1* is a main quantitative trait locus of seed shattering [36]. In addition, another *BLH* gene *SH5* induces seed shattering by facilitating abscission-zone development and inhibiting lignin biosynthesis, and SH5 can interact with KNOX protein OSH15 to induce grain shattering by repressing lignin biosynthesis-related genes [37,38]. One recent study has reported that gene *RI* encoding a BLH transcription factor affects primary branch pattern mainly by regulating the arrangement and initiation time of the primary branch meristems, the *BLH* gene family is essential for regulating inflorescence structure in plant [39]. However, the molecular mechanism by which these genes regulate the branch arrangement pattern remain largely unknown in rice.

In this study, we characterized the rice *verticillate primary branch 1* (*vpb1*) mutant, which displayed a clustered primary branch phenotype. Gene isolation experiment revealed that *VPB1* was a allele of *RI*, and it encoded a BLH transcription factor. Further experiments demonstrated that *VPB1* negatively regulated the expression of *OsBOP1* gene to construct panicle architecture in rice. Transcriptome analysis indicated that *VPB1* was likely to negatively regulate the expression of genes involved in auxin hormonal pathways to form the normal inflorescence architecture. Our results provide new insights into the branching patterns in rice.

## 2. Results

### 2.1. Inflorescence Phenotypes in vpb1 Mutant

**Figure 1:**
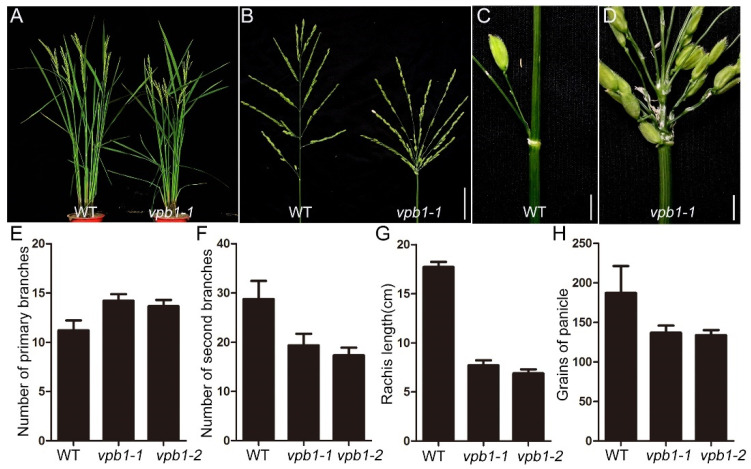
Phenotypic characterization of *vpb1-1* mutant. (**A**) Mature wild-type plants (left) and the *vpb1-1* mutant (right). (**B**) Mature panicles of wild-type (left) and *vpb1-1* mutant (right). (**C**,**D**) Close-up view of the branch site of the primary branches in wild-type (**C**) and *vpb1-1* mutant (**D**). (**E**–**H**) Quantitative traits of wild-type and *vpb1* mutant panicles. Vertical bars indicate standard deviations, *n* = 15. (**E**) The numbers of primary branches in wild type and *vpb1* mutant. (**F**) The numbers of secondary branches in wild type and *vpb1* mutant. (**G**) Rachis length of wild type and *vpb1* mutant. (**H**) The numbers of grains of panicle in wild type and *vpb1* mutant. Scale bars, 4 cm in (**B**); 2 cm in (**C**,**D**).

To identify the key regulators that control panicle architecture formation in rice, we screened two recessive and allelic mutants which exhibited abnormal panicles from rice T-DNA insertion mutant library. We designated them as *verticillate primary branch 1-1 (vpb1-1)* and *vpb1-2* (Figure S1). Compared with wild-type inflorescence, the *vpb1* mutant inflorescence exhibited the clustered primary branch phenotype, indicating the primary branches are initiated in a verticillate manner (Figure 1A–D). Our findings are consistent with a previous report that mutant phenotype of *RI* [39]. To investigate *vpb1* inflorescence quantitatively, we counted the number of inflorescence branches in the wild type and mutant. The primary branches number of *vpb1* mutant panicle was increased by 26.8%, and the secondary branches number was decreased by 32.8%, compared to the wild-type inflorescence (Figure 1E,F). Quantitative analysis of *vpb1* mutant panicle indicated that the length of rachis and the number grains of panicle were respectively reduced by 56.5% and 27% compared with wild types (Figure 1G,H). The clustered panicle appearance and the reduction in spikelet number in the *vpb1* mutant might be attributable to the reduced rachis length and the decreased number of secondary branches. Moreover, the *vpb1* mutants exhibited a defect in producing the inflorescence meristem.

**Figure 2:**
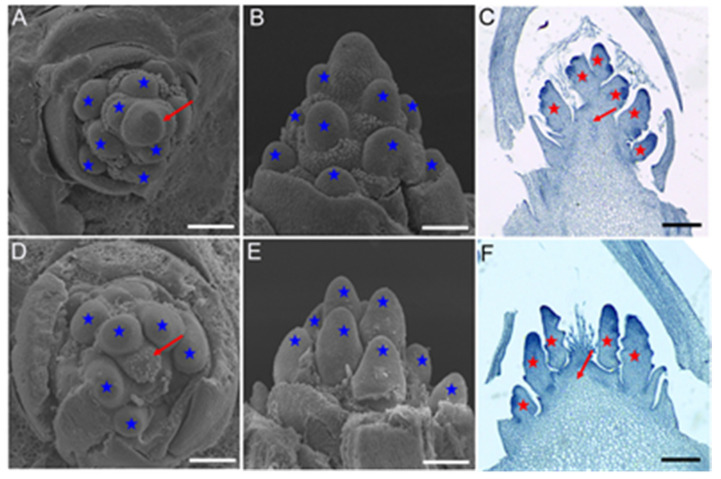
Morphological analysis of wild-type and *vpb1* inflorescence. (**A**,**B**) Scanning electron microscope (SEM) images of PBMs at their initiation stage in wild-type. (**D**,**E**) Scanning electron microscope (SEM) images of PBMs at their initiation stage in *vpb1* mutant. (**C**,**F**) Paraffin section images showing the inflorescence of the wild type (**C**) and *vpb1* (**F**). The arrow and asterisks indicate inflorescence and primary branch meristems, respectively. Scale bar, 100 μm.

To further examine the defects of *vpb1* panicles, we used scanning electron microscope (SEM) to determine the time when the panicle development of *vpb1-1* plants first differed from that wild type plants. SEM results indicated no significant morphological difference between *vpb1* and the wild-type SAMs in the vegetative stage and reproductive stage except the primary branch meristem (PBM) formation stage (Figure 2 and Figure S2). The wild type PBMs were initiated in a regular spiral pattern (Figure 2A). By contrast, *vpb1* mutant PBMs were initiated in an irregular pattern and they might be simultaneously initiated from the inflorescence meristems (Figure 2D). The lateral view of PBMs showed that the height of the PBM cluster of *vpb1* was lower than that of wild type (Figure 2B,E). These results confirmed that the primary branch meristems of *vpb1* mutant displayed an abnormal arrangement on inflorescence meristem. We hypothesized that the disordered primary branch meristems might be caused by the abnormal development of inflorescence. To test this hypothesis, we especially used the paraffin section method to examine morphological characteristics of panicles, we found that the inflorescence meristem of *vpb1* mutant was extremely defective (Figure 2C,F). Therefore, we considered that the disordered phyllotactic pattern of *vpb1* inflorescence might be due to the disturbed arrangement of the primary branch meristems. *VPB1* functioned as a determinant factor to regulate inflorescence meristem activity during panicle morphogenesis.

### 2.2. Map-Based Cloning of VPB1

**Figure 3:**
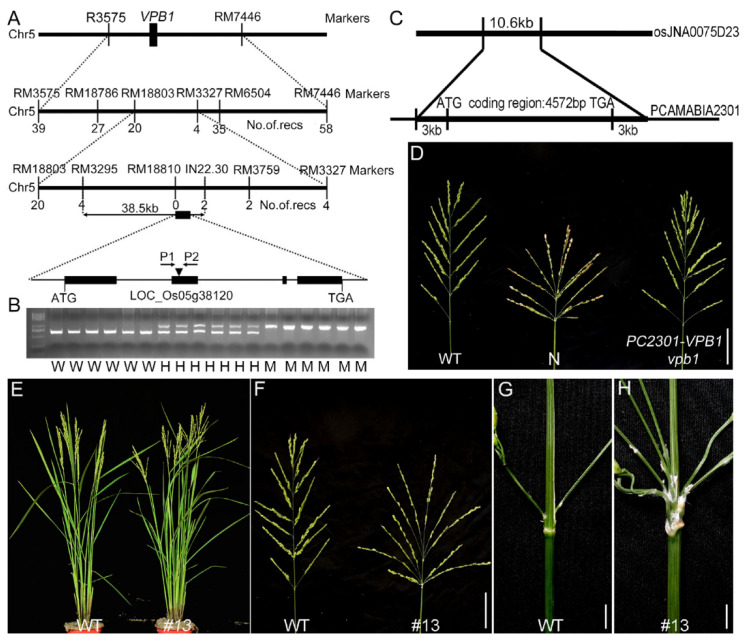
Positional cloning of the gene responsible for the *vpb1* mutation. (**A**) Fine mapping of the *VPB1* on chromosome 5. The *VPB1* locus was narrowed to a ~38.5-kb genomic DNA region between markers RM3295 and IN22.30. recs is the number of recombinants. The structure of *VPB1*, showing the mutation site of *vpb1*. Closed boxes indicate the coding sequence, and lines between boxes represent introns. (**B**) Cosegregation analysis of a F_2_ population derived from a cross of *vpb1* x WT (ZH11) via PCR using the primers (P1, P2) shown in (**A**). M: mutant; H: hetero; W: wild type. (**C**) Schematic diagram of the pC2301-*VPB1* construct. (**D**) Genetic complementation of *vpb1*. N indicates negative control. Scale bar, 4 cm. (E-H) Performance of *VPB1* positive and negative transgenic plants generated using the CRISPR/Cas9 strategy. (**E**) Mature wild-type plants (left) and the *#13* mutant (right). (**F**) Mature panicles of wild-type (left) and *#13* mutant (right). Scale bar, 4 cm. (**G**,**H**) Close-up view of the branch site of the primary branches in wild-type (**G**) and *#13* mutant (**H**). Scale bar, 2 cm.

We constructed a mapping population by crossing the original *vpb1* mutant with *indica* variety Dular. Of 1200 F_2_ plants, 288 exhibited a *vpb1*-like phenotype, and chi-square test results indicated that a segregation ratio of the *vpb1* mutant plants and wild-type plants was 1:3. These results demonstrated that the phenotype of *vpb1* mutant was controlled by a recessive single gene. To clone gene *VPB1* through a map-based approach, Primary gene mapping showed that *VPB1* locus was located between the molecular markers RM3575 and RM7448 on chromosome 5, and we then fine-mapped the locus to a 38.5-kb region between markers RM3295 and IN22.30 (Figure 3A). Within this region, five genes were predicted in the Nipponbare genome (TIGR Rice Genome Annotation Database) (Table S1). PCR-based sequencing and bioinformatics analyses of this 38.5-kb region fragment revealed that a 433-bp DNA fragment was inserted into the second exon of the candidate gene *LOC_Os05g38120* in *vpb1-1* mutant to generate a premature stop codon, and that a 7-bp nucleotide deletion in the second exon in *vpb1-2* led to amino acid frameshift (Figure S3). *LOC_Os05g38120* composed of four exons and five introns encoded a homeodomain protein (Figure 3A and Table S1). To verify whether the clustered primary branch phenotype was caused by the DNA insertion and deletion in *LOC_Os05g38120*, a pair of gene-specific primers P1 and P2 were used to detect the genotype of the F_2_ population derived from the cross of *vpb1* with WT. Cosegregation analysis of an F_2_ population indicated that all the *vpb1-1* plants with homozygous DNA insertion showed the phenotype of the clustered primary branch, and the other plants without DNA insertion or with heterozygous DNA insertion showed normal panicle morphology (Figure 3B), and all the *vpb1-2* plants with homozygous DNA deletion showed the phenotype of the clustered primary branch, and the other plants without DNA deletion or with heterozygous DNA deletion showed normal panicle morphology (Figure S3). Therefore, these results suggested that *LOC_Os05g38120* was determined as the candidate gene of *VPB1*, which was a new allele of *SH5/RI* [37,39].

To test *VPB1* whether could complement the mutant phenotype, we constructed a vector. This vector fragment containing the coding sequence of *VPB1* flanked by a 3000-bp upstream fragment of the start codon and a 3000-bp downstream fragment of the stop codon was cloned into pCAMBIA2301 (Figure 3C). This vector was transformed into *vpb1* mutant callus, and 31 independent transgenic plants were obtained. The abnormal inflorescence phenotype of *vpb1* of these 31 transgenic plants was fully rescued by this constructed pC2301-*VPB1*, whereas that of 12 plants transformed with empty vector (negative control) remained unrescued (Figure 3D). Additonally, we generated function-deficient mutants in the ZH11 background using the CRISPR system (Figure S4) [40], and these mutants displayed reduced rachis length and verticillate primary branches (Figure 3E–H). Afterwards, we transformed vector pC1301S-VPB1-GFP with green fluorescent protein (GFP) fused to the C terminus of VPB1 into rice ZH11 (WT) callus, and obtained multiple independent lines overexpressing *VPB1,* their phenotypes were similar to those of wild-type (Figure S5). Moreover, in the young panicle, the expression of *VPB1* was relatively lowly expressed in mutant, compared to that in wild-type plants (Figure S6A). The immunoblot assay with an anti-VPB1 antibody revealed that the accumulation of VPB1 protein in the young panicle (2–3mm) was greatly reduced in *vpb1-1* and *vpb1-2* (Figure S6B). These results suggested that the mutation of *VPB1* was responsible for abnormal panicle morphology of *vpb1.*

### 2.3. VPB1 Encodes a BELL1-Type Transcription Factor

Bioinformatic analysis revealed that the amino acid sequence of VPB1 contains a conserved BELL domain, indicating that VPB1 is one member of the BLH family. Members of BLH family regulate many key developmental processes in plants [21,27,35,41,42]. Thirteen members of the BLH family have been identified in *Arabidopsis* and 17 members in rice [38]. These BLH proteins domain had three extra amino acids (Proline[P], tyrosine[Y], Proline [P]) between the first and the second helix (Figure S7A). To examine the relationship between VPB1 and other BLH proteins, we used amino acid sequences of VPB1 and other BLH proteins in rice and *Arabidopsis* to construct a phylogenetic tree (Figure S7B). The result revealed that the VPB1 protein was highly homologous to *Arabidopsis* PNY and PNF. Gene *LOC_Os05g38120* has been reported to be *SH5*, phylogenetic analysis also revealed that the VPB1 was highly homologous to qSH1, and that both SH5 and qSH1 were responsible for the formation of seed abscission layer in rice [36,37]. Moreover, the alignment and motif analysis of VPB1 homologue in rice and *Arabidopsis* showed that VPB1 contained the intermediate BLH domain composed of SKY and BELL regions and the C-terminal homeobox domain, and it was relatively conservative in various plant species (Figure S7C).

### 2.4. Expression Pattern of VPB1

**Figure 4:**
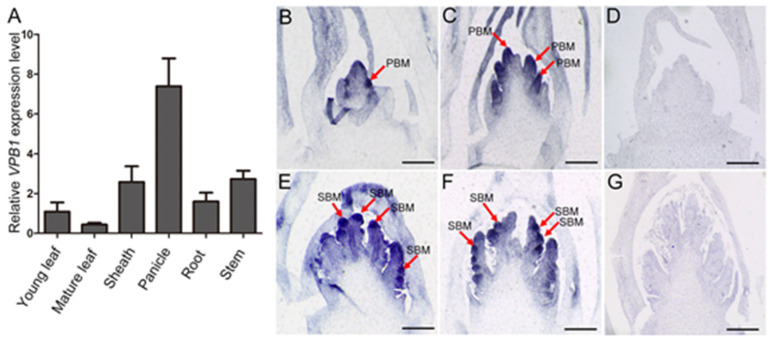
Expression pattern of *VPB1*. (**A**) RT–qPCR of organ-specific *VPB1* expression in WT plants. Including young leaf, mature leaf, sheath, panicle (1–2 mm), root, stem. Data are mean ± SD (n=3 biological replicates). (**B**–**G**) In situ hybridization of *VPB1*. (**B**) Whole a developing inflorescence at the stage of SAM; (**C**) Whole a developing inflorescence at the stage of primary branch meristem (PBM) differentiation; (**E**,**F**) Whole a developing inflorescence at the stage of secondary branch meristem (SBM) differentiation. (**D**,**G**) Sense probe as control. The red arrow points to the branch meristem. Scale bars, 100 μm.

To reveal the role of *VPB1* in inflorescence development, we explored its expression pattern. The qRT-PCR analysis indicated that *VPB1* was expressed in all tested tissues, including young leaf, mature leaf, leaf sheath, panicle, root, and stem; especially, it was expressed more highly in panicle than in other tissues (Figure 4A). RNA in situ hybridization further showed that *VPB1* transcripts were detectable at different inflorescence development stages in wild-type, and that *VPB1* was highly expressed in shoot apical meristem, primary and secondary branch meristem (Figure 4B,C,E,F). This agrees with the results by Ikeda et al. (2019) [39]. As expected, *VPB1* expression was hardly detectable when sense probe was used as a negative control (Figure 4D,G). The expression pattern analysis of both *VPB1* suggested that the *VPB1* gene played a critical role in establishing and maintaining meristem in rice.

### 2.5. Subcellular Localization and VPB1 Transcriptional Activity

**Figure 5:**
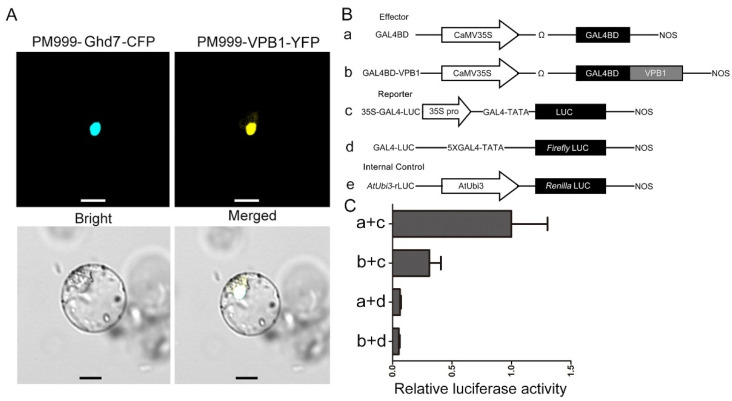
Subcellular localization and transcription activity of VPB1. (**A**) Subcellular localization of VPB1 protein. The VPB1-YFP fusion protein co-localized with the Ghd7 nucleus marker in rice protoplasts. Scale bars, 10 μm. (**B**) Scheme of the constructs used in the protoplast co-transfection assay. (**C**) Transcriptional activity assay of *VPB1*. The activity of 35S-GAL4-LUC and GAL4-LUC was used as the reporter, and rLUC activity was used as an internal control. The fLUC/rLUC ratio represents the relative luciferase activity. Data are mean ± SD (*n* = 3 independent replicates).

Consistent with the function of VPB1 as a transcription factor, through the subcellular localization prediction tool Plant-mPLoc [43], VPB1 was predicated to be located in the nucleus. To test this prediction, VPB1 was fused with YFP, and Ghd7 (a nuclear protein) was fused with cyan fluorescent protein (CFP). The obtained two fusion plasmids were transiently expressed in rice protoplasts, and the fluorescence signal assay indicated that VPB1 and Ghd7 were co-localized to the nucleus (Figure 5A), suggesting that VPB1 was a nuclear protein. 

We next investigated the transcriptional activity of VPB1 using a dual-luciferase reporter system. We constructed an effector GAL4BD-VPB1, and the firefly luciferase gene driven by CaMV35S enhancer contained five copies of the GAL4 binding element, and it was used as a reporter, and the renilla luciferase gene driven by a *AtUbi3* promoter was used as the internal control (Figure 5B). The results showed that the effector GAL4BD-VPB1 had significantly lower relative luciferase activity than the GAL4BD, but no significant difference in relative luciferase activity was observed between the reporter GAL4-fLUC and the GAL4BD (Figure 5C). Based on this result, we concluded that VPB1 could actively mediate transcriptional repression.

### 2.6. VPB1 Affects the Expression of Genes Involved in Inflorescence Development and Hormone Pathways

**Figure 6:**
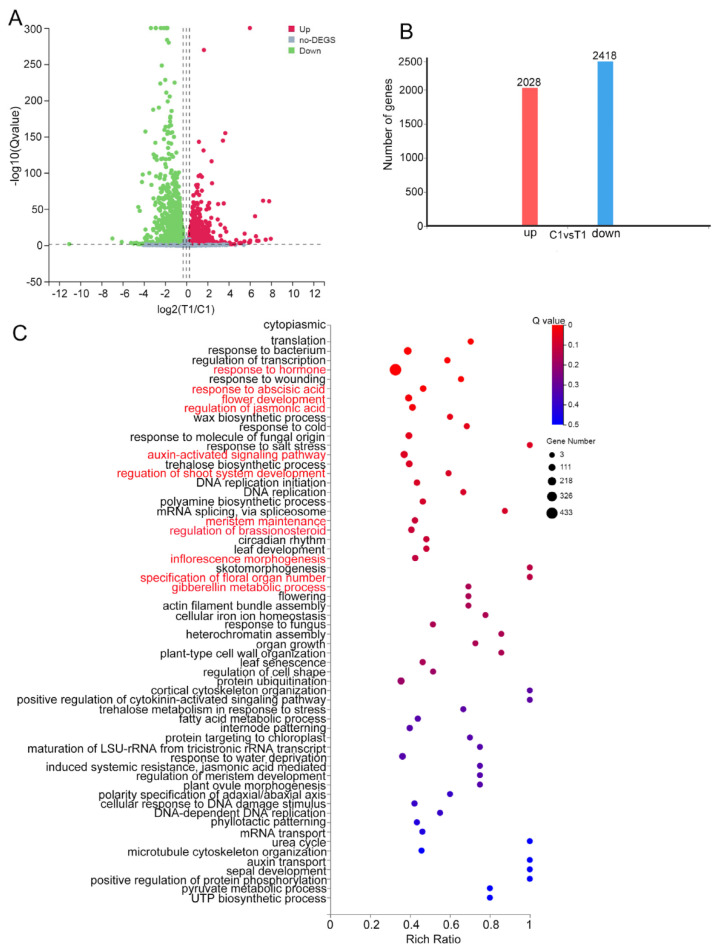
Differentially expressed genes statistics and GO analysis. (**A**) Volcano plots were used to visualize RNA sequencing (RNA—Seq) data. Each point corresponds to a reference sequence (Ref Seq) Gene. Red and green represent upregulated and downregulated genes in *vpb1* lines compared with WT. T1: Mutant treatment group, C1: Wild type control group. (**B**) Statistics of the number of differentially expressed genes. Red represents upregulated DEGs, blue represents downregulated DEGs. (**C**) Gene ontology (GO) analysis functional categories of genes that differed in abundance between *vpb1* and WT. Biological pathways related to hormone signaling and inflorescence architecture are indicated on the left and marked in red. Points of different color and size represent gene numbers.

To reveal the molecular mechanism of inflorescence development in *vpb1* mutant, we analyzed gene expression levels in the young panicle (1–2 mm) of *vpb1-1* mutant and wild type plants at the stage of PBM initiation by RNA-Seq with Q value ≤ 0.05 and fold change ≥ 1.5 as the cutoff criteria. We identified differentially expressed genes (DEGs) between wild type and mutants in three biological replicates. A total of 2028 genes were upregulated, and 2418 genes were downregulated in *vpb1-1* mutant, compared with wild type (Table S2 and Figure 6A,B). Further gene ontology (GO) analyses revealed that these DEGs were enriched in multiple biological processes, including transcription regulation, plant hormone signal transduction, flower development, shoot system development regulation, meristem maintenance, internode patterning, organ growth, and metabolism processes (Figure 6C), suggesting that *VPB1* participated in a complex regulation network of rice inflorescence development.

**Figure 7:**
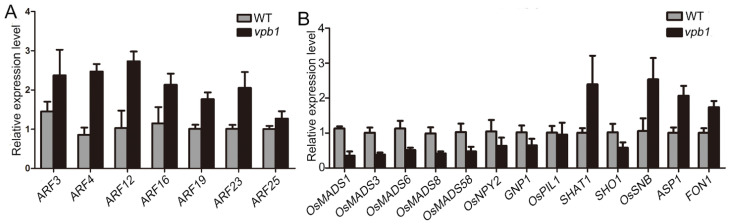
*VPB1* regulates genes involved in hormone signaling and meristem maintenance. (**A**) Expression analysis of the auxin responsive factor genes in WT and *vpb1* young panicles. Data are mean ± SD. (*n* = 3 biological replicates). (**B**) Expression analysis of the related to meristem maintenance genes in WT and *vpb1* young panicles. Data are mean ± SD. (*n* = 3 biological replicates).

Auxin signaling and transport have been reported to be important determinants of inflorescence development in *Arabidopsis* [34]. Our DEG analysis revealed that *VPB1* mainly participated in the auxin pathway and affected the genes related to meristem activity and inflorescence development. For example, genes *OsMADS1*, *OsMADS3*, *OsMADS6*, *OsMADS8*, and *OsMADS58* have been reported to interact with each other to promote flower development, which is very important for the maintenance of flower meristem identity and the formation of flower organ [44], and genes *GNP1*, *OsNPY2*, *SHAT1*, *FON1*, *ASP1*, *SHO1*, *OsSNB*, and *OsPIL1* are associated with the abscission zone development, meristem activity and fate, internode patterning, and inflorescence morphology [18,45,46,47,48]. To verify RNA-seq results, qPCR was used to analyze auxin pathway-related 7 genes and the above-mentioned 15 genes in the young panicle (2mm) of WT and *vpb1* plants. Our data indicated that the results of RNA-seq and qPCR were consistent, seven *ARFs* genes in the auxin pathway were strongly upregulated in *vpb1* mutant at young panicle stage (Figure 7A), and *MADS-box* genes and eight genes mainly involved in the maintenance of meristem activity were significantly different between wild type and *vpb1* mutant (Figure 7B). Taken together, RNA-seq results indicated that *VPB1* ensured the formation of normal panicle architecture by regulating the expression of the genes related to auxin pathways and inflorescence meristem development.

Results indicated that *VPB1* suppressed the expression of *OsBOP1*. 

### 2.7. VPB1 Negatively Regulates OsBOP1 Expression

**Figure 8:**
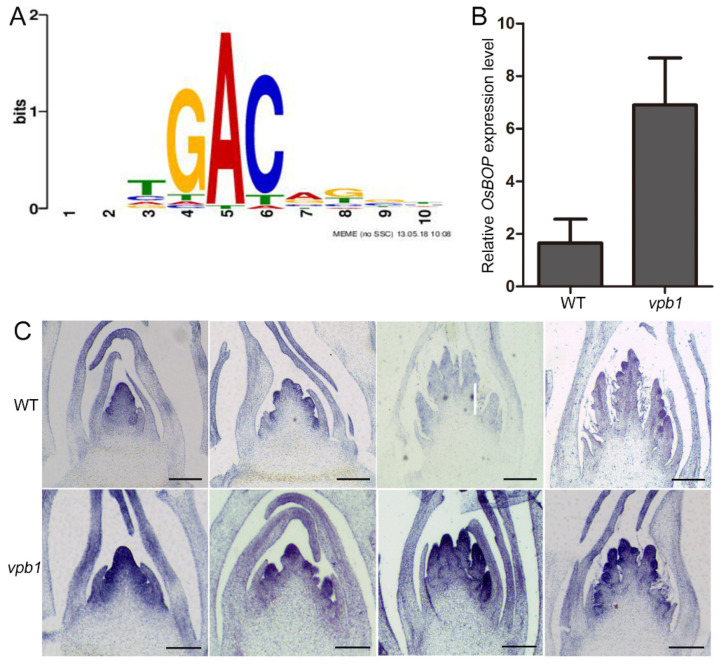
*VPB1* negatively regulates *OsBOP1* expression. (**A**) Putative TFBS for VPB1. (PlantPan3.0: http://plantpan.itps.ncku.edu.tw/index.html (accessed on 21 November 2020). (**B**) RT–qPCR analysis of *OsBOP1* expression in WT and *vpb1* young panicles (2 mm). Data are mean ± SD. (*n* = 3 biologically independent replicates). (**C**) In situ hybridization of *OsBOP1* mRNA in WT and *vpb1* in different stages of inflorescence development. Scale bars, 100 μm.

Evidence suggests that the BEL-type proteins regulate downstream target gene transcription by recognizing a core motif of these genes’ promoters in *Arabidopsis* [49]. In our study, VPB1 encoded BLH proteins belonging to TALE family. Thus, to identify potential target genes of VPB1 protein, we downloaded TALE family binding core motifs (TFmatrixID_0278, Figure 8A) from PlantPAN 3.0 website [50], and we screened RNA-seq-obtained DEGs containing the core motifs from the upstream 2 kb promoter regions of DEGs with MEME FIMO [51]. The results revealed that a total of 682 DEGs with core motifs were screened, including 309 upregulated and 373 downregulated genes (Table S3). Since *VPB1* was transcriptional repressor, we further analyzed these 309 upregulated genes, and we found that genes *OsBOP* genes were related to meristem development. Therefore, we speculated that *OsBOPs* might be a potential target gene of *VPB1*.

To test whether the *OsBOP* expression was directly regulated by VPB1 protein, we first compared the expression patterns of *OsBOP1* in WT and *vpb1*. The qRT-PCR analysis revealed that in young panicle (1–2 mm), the *OsBOP1* expression level was higher in *vpb1* than in WT (Figure 8B). Consistently, in situ hybridization experiments detected a broader expression of *OsBOP1* in the SAM, PBMs and SBMs in *vpb1* mutant plants than in WT plants with its expression expanded throughout the PBMs and SBMs (Figure 8C). These 

**Figure 9:**
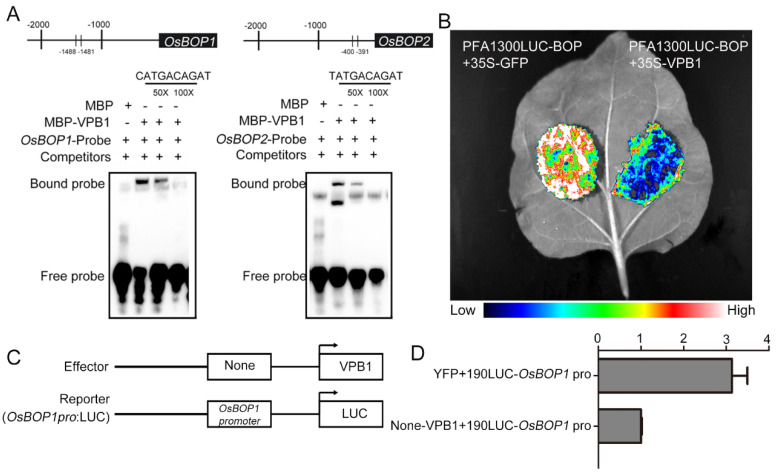
VPB1 is the transcriptional repressor of *OsBOP1*. (**A**) Schematic diagram of the OsBOP1/2 promoter showing the potential VPB1 binding sites and EMSA of MBP and MBP—VPB1 recombinant proteins incubated with biotin—labeled probes of *OsBOP1* and *OsBOP2*. Numbers above the diagram indicate the distance away from ATG. Competition for binding was performed using 50× and 250× competitive probes; MBP was used as a negative control. (**B**) Analysis of the binding ability of VPB1 with the *OsBOP1* promoter transiently expressed in tobacco leaves by transient expression regulation assays, showing that VPB1 protein suppresses the expression of *OsBOP1*. (**C**) Scheme of the constructs used in the protoplast dual luciferase reporter assays. (**D**) Dual luciferase reporter assays in rice protoplasts shows that the VPB1 protein suppresses the expression of LUC gene through binding to the *OsBOP1* promoter. Data are mean ± SD (*n* = 3 independent replicates).

Then, we examined the ability of VPB1 protein to bind to the promoter region of *OsBOPs* using electrophoretic mobility shift assay (EMSA). Promoter analysis revealed that *OsBOPs* contained TALE family core motif (Table S3). Thus, *OsBOP1* promoter fragment (50 bp) containing the 10-bp sequence CATGACAGAT and *OsBOP2* promoter fragment (50 bp) containing the 10-bp sequence TATGACAGAT were selected for EMSA. We constructed MBP protein and MBP-VPB1 fusion protein, and by using them, we detected the shifted bands which combined MBP-VPB1 fusion protein and the probes with CATGACAGAT and TATGACAGAT in the *OsBOPs* promoter region, but not the shifted bands of MBP protein (Figure 9A). 

Additionally, we attempted to confirm VPB1 binding ability in *Nicotiana benthamiana* leaves using transient expression assays. Strong signals were detected in tobacco leaves when pro*OsBOP1*: LUC was transformed, but only weak signals were detected when VPB1 protein was coexpressed with pro*OsBOP1*: LUC (Figure 9B). This result indicated that VPB1 could directly bind to the *OsBOP1* promoter to repress its expression. Finally, dual luciferase reporter assays in rice protoplasts showed that VPB1 could suppress the expression of LUC gene by binding to the *OsBOP1* promoter (Figure 9C,D). In addition, we created a double mutant *vpb1/osbop1*, and found that the morphology of *osbop1* single mutant plants was normal, but the *vpb1/osbop1* double mutant plants exhibited similar phenotype with the *vpb1* mutant plant, indicating inflorescence architecture defects caused by *vpb1* mutation were not rescued (Figure S8). Importantly, our data demonstrated that *VPB1* controled the inflorescence development by directly negatively regulating the expression of *OsBOP* genes.

## 3. Discussion

### 3.1. VPB1 Regulates the Initiation and Arrangement of Primary Branch Meristems

The normal development of the primary branch meristems is important for the inflorescence architecture of rice [8]. Morphological analysis at the stage of primary branch development indicated that in *vpb1* mutant plants, the initiation timing and arrangement of the primary branch meristems were abnormal, that inflorescence meristem was damaged, and that the activity of the inflorescence meristem was reduced, resulting in the clustered primary branch meristems, but the secondary branch meristems and spikelets were less affected, suggesting that *VPB1* mainly maintained the activity of inflorescence meristem and regulated the phyllotactic pattern of the primary branches. Similarly, we found that *VPB1* was expressed in shoot apical meristem in the early stage of panicle development, and specifically expressed in the primary and secondary branch meristems. Moreover, *PNY* gene is essential for the formation of meristems and the maintenance of activity in *Arabidopsis* [23]. Collectively, these observations indicated that *VPB1* gene ensured the initiation and fine arrangement of the branch meristem by maintaining the activity of the inflorescence meristem.

### 3.2. VPB1 Belongs to a Functionally Conserved Gene Family

Numerous previous studies have reported that *BLH* genes influence many aspects of plant morphology and typically maintain the meristem activity essential for organ formation [21,35,39,42], but little is known about their involvement in the regulation of panicle morphology development in rice. In this study, we isolated the key regulator *VPB1* encoding a BLH protein, and we found that the functional loss of *VPB1* resulted in clustered primary branches and short rachis. Thus, it could be concluded that *VPB1* played an important role in inflorescence formation. Positional cloning revealed that *VPB1* was identical to the previously reported *RI* gene which was identified as the ortholog of *Arabidopsis* *PNY* and maize *BLH12/14*, indicating that *VPB1* was involved in forming normal inflorescence architecture by regulating the phyllotactic pattern [30,35,39]. These findings supported that the BLH transcription factors had partially conserved functions in regulating the inflorescences in dicots and monocots.

### 3.3. The VPB1 Gene Participates in a Complex Molecular Pathway to Regulate Panicle Development

TALE genes are well-known to play critical roles in regulating inflorescence architecture by affecting plant hormones [21]. For example, in *Arabidopsis*, *PNY* has been reported to directly target the auxin transport- and signaling pathway- related genes [34]. The mutually combined transcriptional regulators ETT, IND, BP, RPL, and SEU regulate the transcription of genes responsible for inflorescence development and auxin polar transport to facilitate proper auxin distribution in inflorescence in brassicaceae [52]. Our RNA-seq results showed that VPB1 was a powerful regulatory protein, and it significantly affected the genes related to the auxin, brassinosteroid (BR), abscisic acid, and gibberellin pathways (Figure 6C). Interestingly, *CPB1* (a new allele of *D11*) has been reported to encode a cytochrome protein P450 which is involved in BR biosynthesis pathway, and *cpb1* mutant plants also exhibit a clustered primary branch phenotype, compared to wild type plants [53]. Therefore, we guessed that *VPB1* might regulate the expression of *CPB1* gene during inflorescence development. We further analyzed the expression levels of auxin-related genes (*ARFs)* in WT and *vpb1* young panicles by qRT-PCR (Figure 7A). Our qRT-PCR results were consistent with RNA-seq data. Based these results, we speculated that the distribution or content of auxin in the *vpb1* mutant has changed, reducing the activity of the inflorescence meristem, and ultimately leading to the disorder of the initiation and arrangement of the branch meristem, the mechanism underlying *VPB1* regulation of branch arrangement in relation to auxin action is important issues to be resolved in our future studies. 

Our data indicated the phenotype of the *vpb1* mutant plant might be caused by the reduced inflorescence meristem activity. Notably, our DEG analysis revealed that *VPB1* regulated multiple genes involved in the meristem identity maintenance and inflorescence development. The expressions of these genes exhibited significant difference between wild type and *VPB1* mutant (Figure 7B). The possible reason for such difference might lie in that the *VPB1* made these genes unable to be normally expressed in meristems, thus causing the failure in maintaining inflorescence meristem growth. Alternatively, the inhibition of inflorescence meristem activity might be associated with a change in cell wall components, as reported in *Arabidopsis* [31]. The regulation mechanism by which the change in cell wall components affects meristem activity remains to be further investigated in future studies.

### 3.4. VPB1 Regulates Inflorescence Development by Directly Binding to OsBOP1

This study indicated that *VPB1* was a transcriptional repressor. Our RNA-seq data of *vpb1* young panicle revealed that a total of 2028 genes were upregulated (Table S2). Of these upregulated genes, some genes were found to contain the conserved TALE core motifs, such as *OsBOP* genes. Previous studies have shown that *BOP1* and its highly homologous gene, *BOP2*, are involved in floral patterning, abscission zone formation, and bract suppression, and control of axillary bud growth and inflorescence development in plants [54,55,56]. Three *BOP* genes (*OsBOP1*, *OsBOP2,* and *OsBOP3*) in rice determine the leaf sheath: blade ratio by activating proximal sheath differentiation and suppressing distal blade differentiation, and these three genes are related to the microRNA156/SPL pathway [57]. Pioneering work in *Arabidopsis* has shown that *PNY* directly binds to *BOP1*, *BOP2,* and *KNAT6* to inhibit their expressions, eventually to regulate inflorescence development [49,58]. Our dual-luciferase reporter system and EMSA confirmed that the expressions of these genes were repressed by VPB1, and that the expression level of *OsBOP1* involved in the boundary organ initiation pathway was significantly upregulated in *vpb1* mutant young panicle. Consistently, RNA in situ hybridization assay indicated that *VPB1* suppressed expression of *OsBOPs*. However, inflorescence architecture defects caused by *vpb1* mutation were not rescued in the *vpb1/osbop1* double mutant plants. Previous research has shown that *OsBOP* genes in rice redundantly control leaf development [57]. Considering this, we speculated that three *OsBOP* genes might also redundantly control inflorescence architecture, in addition to regulating *OsBOP1*, *VPB1* might also regulate other downstream target genes to control panicle development. Based on these findings, it could be concluded that VPB1 protein could directly interact with the promoter of these *OsBOP* genes and suppress their transcriptions to maintain the normal development of inflorescence meristem. The genetic relationship between *VPB1* and *OsBOP* genes will be the focus of our future research. 

### 3.5. The Role of BLH-KNOX Dimer Functions in Inflorescence Development 

The interaction between BLH and KNOX homeobox proteins to form heterodimers has been widely reported [32,59], and these two proteins can form complexes and participate in meristem maintenance and the plant growth and development regulation. For example, PNY physically interacts with BP to form BP-PNY complex required by normal inflorescence architecture development [30]. Our study found VPB1 interacted with OSH1 and OSH15 (Figure S9), which are consistent with previously reported that SH5 can interact with OSH15 protein [38]. These findings indicated that the mechanism by which *BLH* and *KNOX* transcription factors regulated inflorescence architecture in rice was similar to that in *Arabidopsis*. Overall, VPB1 interacted with the typical genes of the KNOX family OSH1/OSH15 to form a protein complex, thus regulating panicle architecture development in rice.

Notably, the *vpb1* mutant identified in this study represents a new allele of the rice gene *SH5* regulating seed shattering [37,38]. Based on these results, we speculated that the *BLH* genes may play different roles and participate in different biological processes across rice varieties. Thus, identifying favorable alleles of *VPB1* will enrich our knowledge of panicle architecture in rice.

## 4. Materials and Methods

### 4.1. Plant Materials and Growth Conditions

This study used *Oryza sativa* subspecies *Japonica* “Zhonghua11” (ZH11) as rice materials. The mutant *vpb1* was derived from our T-DNA insertional mutant library (http://rmd.ncpgr.cn/ (accessed on 13 July 2016)). Plants were cultivated under natural long day (LD) conditions during the rice growing season in the experimental field of Huazhong Agriculture University, Wuhan, China, and they were moved to a greenhouse during the winter. All transgenic plants were grown under similar growth conditions.

### 4.2. Scanning Electron Microscopy

In scanning electron microscopy assay, young panicles from WT and *vpb1-1* mutants at different developmental stages were dissected, and immediately fixed in solution containing 70% ethanol, 3.7% formaldehyde, and 5% acetic acid for 24 h at 4 °C overnight. Tissues were dehydrated with a concentration series of ethanol from 25% to 100% and air-dried. After ethanol dehydration and drying, the samples were coated with gold by using an E-100 ion sputter, and then observed under a scanning electron microscope (S570, Hitachi, Tokyo, Japan).

### 4.3. Histological Sectioning

For paraffin sectioning, young panicles from wild type plant and *vpb1-1* mutant plant at different developmental stages were dissected. The samples were fixed in FAA solution at ratio of formaldehyde: glacial acetic acid: ethanol = 1:1:18, v/v/v at 4 °C for 24 h. Subsequently, the samples were dehydrated and cleared in a graded series of ethanol and xylene. The samples were microtome sectioned at the thickness of 5 μm. Afterwards, the sections were stained with 0.5% toluidine blue at room temperature for 30 min, and they were observed with a light microscope.

### 4.4. Map-Based Cloning of VPB1

To determine the *vpb1* locus, we crossed the *vpb1* mutant with *indica* variety Dular to obtain F_1_ plants, and generated an F_2_ mapping population through F_1_ self-crossing. For rough mapping, 15 F_2_ *vpb1* plants and 15 WT plants were used to establish two DNA pools. A total of 1200 independent individuals from the F_2_ population were adopted for fine mapping. The five genes were screened from 38.5 kb regions between two genetic markers on the physical map. Genotyping analysis of the *vpb1* co-segregating population was performed by PCR with the primers *VPB1*-CS-P1 and *VPB1*-CS-P2. PCR was conducted as follows: pre-denaturation at 95 °C for 5 min, followed by 32 cycles of denaturation 95 °C for 45 s, annealing at 58 °C for 45 s, and extension at 72 °C for 1 min. Subsequently, PCR products were verified by sequencing.

### 4.5. Plasmid Construction and Rice Transformation

To prepare the complementation vector, we extracted ZH11 BAC clone OSJNA0075D23, and used PCR to amplify this clone into three fragments and obtained a about 10.6 kb foreign fragment consisting of the entire *VPB1* gene coding region, one 3 kb fragment in front of the ATG, and another 3 kb fragment behind the stop code. We connected this foreign fragment to the PCAMBIA2301 vector by the Gibson Assembly Master Mix (NEB, catalog, E2611L). For overexpression of *VPB1*, the full-length cDNA sequence of *VPB1* was amplified with primer pair *VPB1*-OX-F/*VPB1*-OX-R, and then cloned into pCAMBIA1301S by KpnI-XbaI digestion. For overexpression of *OsBOP1*, the full-length cDNA sequence of *OsBOP1* was amplified with primer pair *OsBOP1*-OX-F/*OsBOP1*-OX-R, and then cloned into pCAMBIA1301S by KpnI-BamHI digestion. Two 20-bp fragments targeting *LOC_Os05g38120* were designed to generate *VPB1* knockout mutants by using CRISPR/Cas9 vector system [40]. The target fragment was inserted into the binary vector pYLCRISPR/Cas9-MH. The above constructs were introduced into *Agrobacterium tumefaciens* EHA105 and homozygous callus from *vpb1* mutuant plant and wild type plant (ZH11), as previously reported [60]. All the primers were listed in Table S4.

### 4.6. Total RNA Isolation and qRT-PCR Analyses

Total RNA was extracted with TRIzol reagent (Invitrogen, Shanghai, China). The 3 μg of RNA was treated with RNase-free DNaseI (Invitrogen). Subsequently, we synthesized first-strand cDNA with oligo (dT)18 primer (TaKaRa, Kyoto, Japan) and M-MLV reverse transcriptase (Invitrogen, Shanghai, China). The qRT-PCR was performed with SYBR Green Master MIX (Roche) in a total 10 μL reaction system on the Applied Biosystems ViiA 7 Real-Time PCR system according to the manufacturer’s instructions. Data were normalized into the internal rice *ubiquitin* (*UBQ*) gene. The relative quantification method (2(-Delta Delta CT)) was used for data analysis. All primers were listed in Table S4.

### 4.7. In Situ Hybridization

Sample fixation and sectioning were performed as described above, followed by hybridization and immunological detection in the previously reported method [61]. The gene-specific primers were used to amplify the probes of *VPB1*, *OSH1*, and *OsBOP1* by PCR. The forward and reverse primers were fused with T7 and SP6 promoters, respectively. SP6 and T7 RNA polymerases were used to transcribe the antisense and sense probes in vitro, respectively, using the digoxigenin-labeled nucleotide mixture (Sigma-Aldrich, St. Louis, MO, USA).

### 4.8. Subcellular Localization 

To construct the subcellular localization plasmids, primers *VPB1*-pM999-F and *VPB1*-pM999-R with KpnI-XbaI digestion sites were used to amplify the full-length cDNA of *VPB1,* and then amplified product was inserted into pM999-YFP vector. The obtained constructs were transformed into rice protoplasts isolated from two weeks etiolated seedlings and incubated at 23 °C for 12 ± 16 h. After incubation, the fluorescence of transformed protoplasts was observed with a confocal laser scanning microscope (TCS SP2; Leica, Weztlar, Germany). 

### 4.9. Transcriptional Activity Analysis

Dual-Luciferase Reporter assay system (Promega, Madison, WI, USA) was used to analyze the transcriptional activity of VPB1 in rice protoplasts prepared from etiolated seedlings [62]. We used the GAL4-responsive vector as a reporter, which was produced by fusing the firefly *LUC* gene driven by the CaMV 35S promoter, five copies of the GAL4 binding site in tandem, and a minimal TATA box, and used the *Renilla luciferase* gene driven by *Arabidopsis thaliana UBIQUITIN3* promoter as internal control. The full-length coding sequence of *VPB1* was amplified using the primers GAL4BD-VPB1-F and GAL4BD-VPB1-R (Table S4) with EcoRI-SalI sites, and the amplified product was inserted into the vector that contained GAL4BD where it acted as an effector. In each transcriptional activity assay, we co-transformed the reporter, effector, and internal control into rice protoplasts in a ratio of 5:5:1 and incubated them at 23 °C for 12 ± 16 h. After incubation, the relative luciferase activity was measured in the DLR assay system with the TECAN Infinite M200 microplate reader.

To assess the specific binding ability of *OsBOP1* promoter, we prepared rice protoplasts from two-week-old fully green plant of ZH11 variety [63]. We inserted the coding sequence of *VPB1* into the NONE vector with the EcoRI-SalI sites to obtain an effector plasmid. Then, we amplified a 2000-bp upstream fragment of the *OsBOP1* promoter, and inserted the amplified product into 190-LUC vector with the HindⅢ sites to construct the *OsBOP1*: LUC reporter vector. The *Renilla luciferase* gene driven by CaMV 35S was used as internal control. In each transcriptional activity assay, we co-transformed 5 μg of effector plasmid DNA and 5 μg of reporter plasmid DNA into rice protoplasts. All primers were presented in Table S4.

### 4.10. RNA-Seq Analysis

We isolated total RNA from 2 mm young panicles of WT plants and vpb1 mutant plants. The experiment had three biological replicates. RNA-seq library was constructed and sequenced using DNBSeq at the Wuhan Genome Institute (BGI) (China). The clean reads were mapped to the rice reference genome (Os-Nipponbare-Refrence-IRGSP-1.0, MSU7) using Hisat2 (http://ccb.jhu.edu/software/hisat2/index.shtml (accessed on 27 October 2020). Q value ≤ 0.05 and fold-change (|Log2 ratio|) >1.5 were considered as statistically significantly different. The GO analysis of DEGs was performed using agriGO [64]. 

### 4.11. EMSA

Promoter *OsBOP1* with core motif CATGACAGAT and promoter *OsBOP2* with core motif TATGACAGAT were produced by annealing oligonucleotides with biotin 5’-end labeled *OsBOP1*-EMSA-F/R, *OsBOP2*-EMSA-MF/MR, respectively. In each reaction, we incubated 50 fmol biotin-labeled probes with the MBP-VPB1 protein in the binding buffer containing 10 μMZnCl_2_, 10 mM Tris, 50 mM KCl, 1 μg/μL poly (dI-dC), 1 mM DTT, 0.05% NP-40, and 0.1% BSA, 2.5% glycerol on ice for 30 min by using the LightShift Chemiluminescent EMSA kit. EMSA was performed as previously reported [61].

### 4.12. The Transient Expression System in Tobacco

To construct the tobacco transformation plasmids, primers PFA1300-BOP1-F/R with KpnI-SalI digestion sites were used to amplify a 2000-bp upstream fragment of the *OsBOP1* promoter*,* and then amplified product was inserted into PFA1300-LUC vector, primers 35S-GFP-VPB1-F/R with KpnI-BamHI digestion sites were used to amplify the full-length cDNA of *VPB1,* and then amplified product was inserted into 35S-CGFP vector. The vector combination 35S-CGFP-VPB1/PFA1300-LUC-BOP1, 35S-CGFP/PFA1300-LUC-BOP1 were transformed into *Nicotiana benthamiana* leaves by *Agrobacterium*, repeated three times, cultured at room temperature for two days, and the whole leaves were injected with 1 mM luciferase substrate. Images were visualized on Tanon-5200 Chemiluminescent Imaging System (Tanon Science and Technology).

### 4.13. Accession Numbers

Sequence data used in this study were downloaded from the Rice Genome Annotation Project website (MSU) and TAIR library. The accession numbers of genes were as follows: *VPB1* (*LOC_Os05g38120*), *qSH1* (*LOC_Os01g62920*), *OSH1* (*LOC_Os03g51690*), *OSH15* (*LOC_Os07g03770*), *OSH71* (*LOC_Os05g03884*), *OsBOP1* (*LOC_Os01g72020*), *OsBOP2* (*LOC_Os11g04600*), *OsBOP3* (*LOC_Os12g04410*), *PNY* (*AT5G02030*), *BOP1* (*AT3G57130*), and *BOP2* (*AT2G41370*).

## 5. Conclusions and Future Prospects

In conclusion, we show that a BELL-like homeodomain protein, VPB1, is involved in the regulation of panicle architecture in rice. *VPB1* loss-of-function mutants exhibited the clustered primary branch phenotype and the length of rachis was reduced. Map-based cloning revealed that *VPB1* is identical to previously reported *SH5/RI* gene [38,39]. While the *SH5/RI* gene and its protein as an interactor with KNOX protein with key roles in rice panicle development has been reported in gene expression studies, few studies provided a molecular mechanism of the panicle branching patterns in rice based on mutant analysis. This study fills that gap in knowledge and provides evidence that *VPB1* regulates the expression of related genes involved in inflorescence meristem development and auxin pathways, and directly inhibits the expression of lateral organ gene *OsBOP1*, maintaining the balance of inflorescence meristem and lateral meristem development, thereby ensuring the fine arrangement of panicle branches. Therefore, these results indicate that *VPB1* is a key gene for the normal arrangement of panicle branches in rice.

Indeed, VPB1 can interact with OSH1 and OSH15 to form heterodimers, indicating that VPB1 may regulate the panicle branching patterns in rice by functioning as heterodimers with KNOX proteins, but few of their functions have been identified. Is it possible to recruit more proteins after the formation of heterodimers to participate in the regulation of panicle development? We hope that their functions can be revealed in our future studies. In addition, VPB1, as a transcription factor with DNA binding ability, obtained 682 genes containing core sequences by analyzing the promoter sequences of the differential genes. What are its downstream target genes besides *OsBOP1* gene? It will be interesting to determine how *VPB1* genetically interacts with these genes to regulate rice panicle morphogenesis in future research.

## Data Availability

The data presented in this study are available on request from the corresponding author.

## Supplementary Materials

The following are available online at https://www.mdpi.com/article/10.3390/ijms22157909/s1.
