# Supplementary material for: VPB1 Encoding BELL-like Homeodomain Protein Is Involved in Rice Panicle Architecture
Source: Int J Mol Sci. 2021 Jul 24;22(15):7909. doi: 10.3390/ijms22157909 (PMC8348756; doi:10.3390/ijms22157909)
Supplement: Supplementary file 1 [file ijms-22-07909-s001.zip › ijms-1280996 suppl figures.pdf]

Supplementary Materials

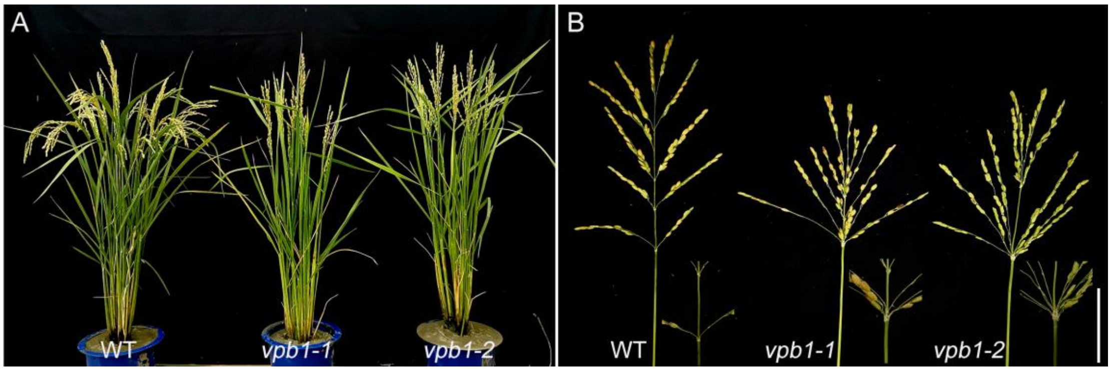

Figure S1. Phenotypic analysis of WT and the *vpb1* mutant. (A) Mature wild-type plants (left) and the vpb1 mutant (right). (B) Mature panicles of wild-type and *vpb1* mutant. Scale bar, 2 cm.

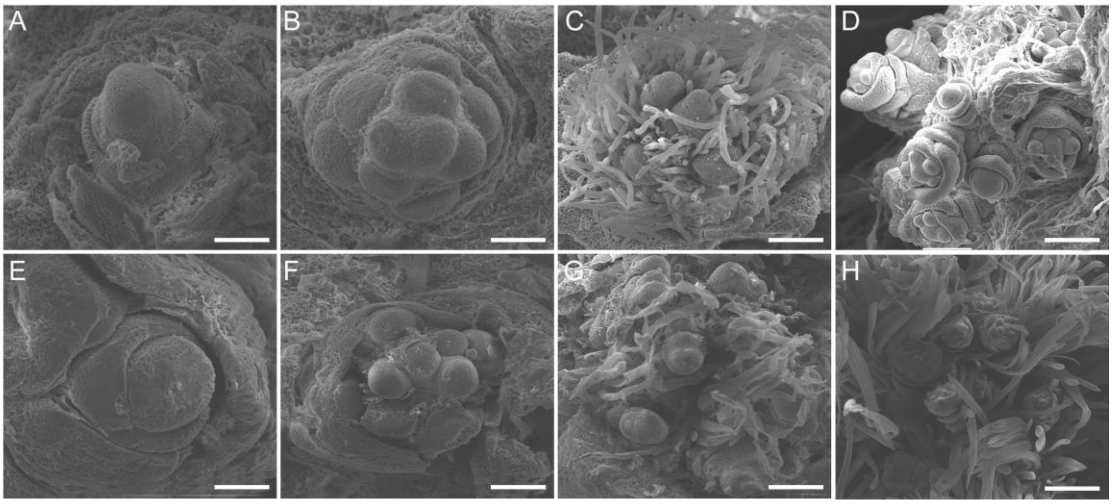

Figure S2. Scanning electron microscope analysis of wild-type and *vpb1* inflorescence. (A, E) Scanning electron microscope (SEM) images, showing the SAM of the wild type (A) and *vpb1* (E) undergoing the transition from the vegetative to the reproductive phase. (B, F) SEM images showing the formation of primary branches of the wild type (B) and *vpb1* (F). (C, G) SEM images showing the formation of secondary branches of the wild type (C) and *vpb1* (G). (D, H) SEM images showing the differentiation of flower organs in the wild type (D) and *vpb1* (H). Scale bar, 100 μm.

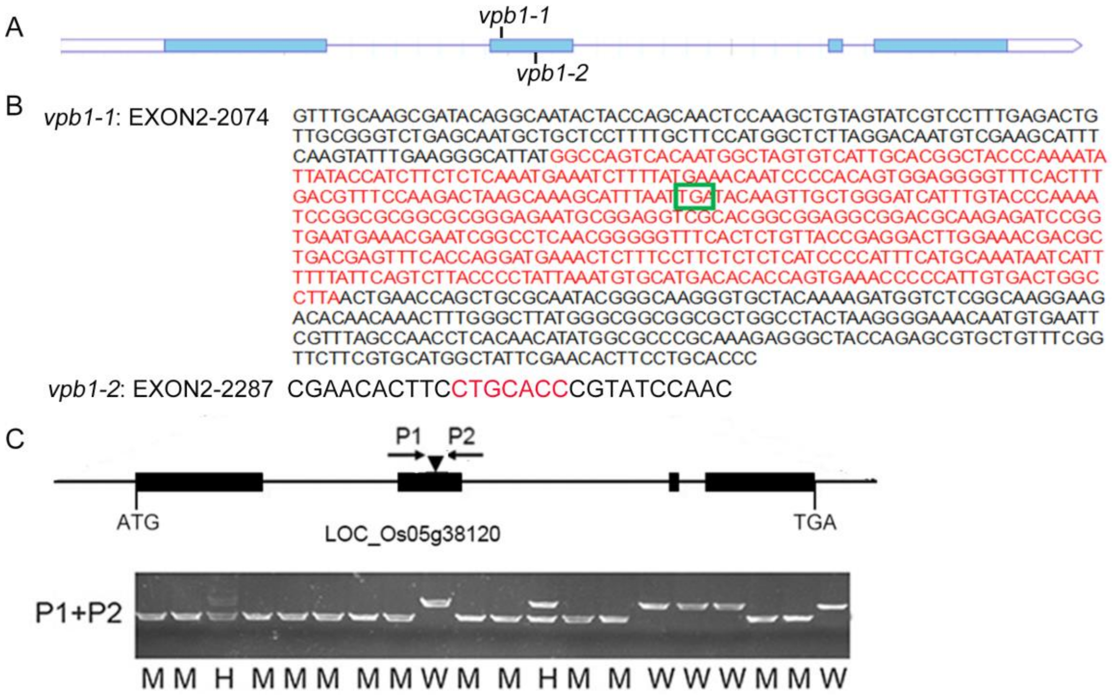

Figure S3. The detailed positions of DNA insertion and deletion sites in *vpb1* alleles and cosegregation analysis. (A) *VPB1* gene structure diagram and *vpb1* alleles mutation sites. (B) *vpb1* alleles insertion and deletion DNA fragment. The red fragments represent insertions and deletions, and the green boxes represent stop codon. (C) Cosegregation analysis of a F_2 population derived from a cross of *vpb1-2* x WT (ZH11).

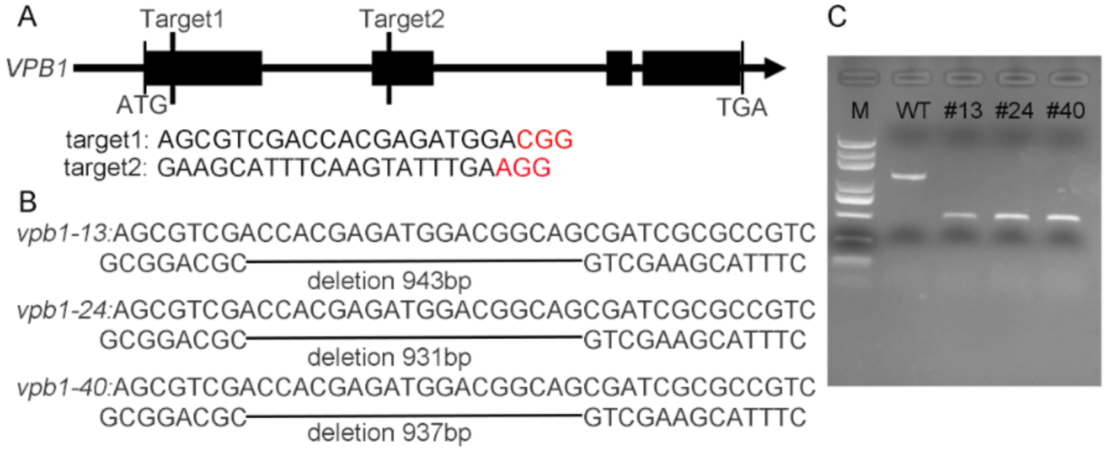

Figure S4. Targeted mutagenesis of *VPB1* genes using the CRISPR-Cas9 system. (A) Schematic map of the genomic region of VPB1 and the sgRNA target sites. The 20-bp target sequences are shown at the bottom of the gene structure, and the following protospacer adjacent motifs (PAMs) (NGG) are labeled in red color. (B) Examples of mutations at the *VPB1* locus in CRISPR-VPB1 T0 generation plants. (C) Gel electrophoresis of PCR products amplified from the mutated region.

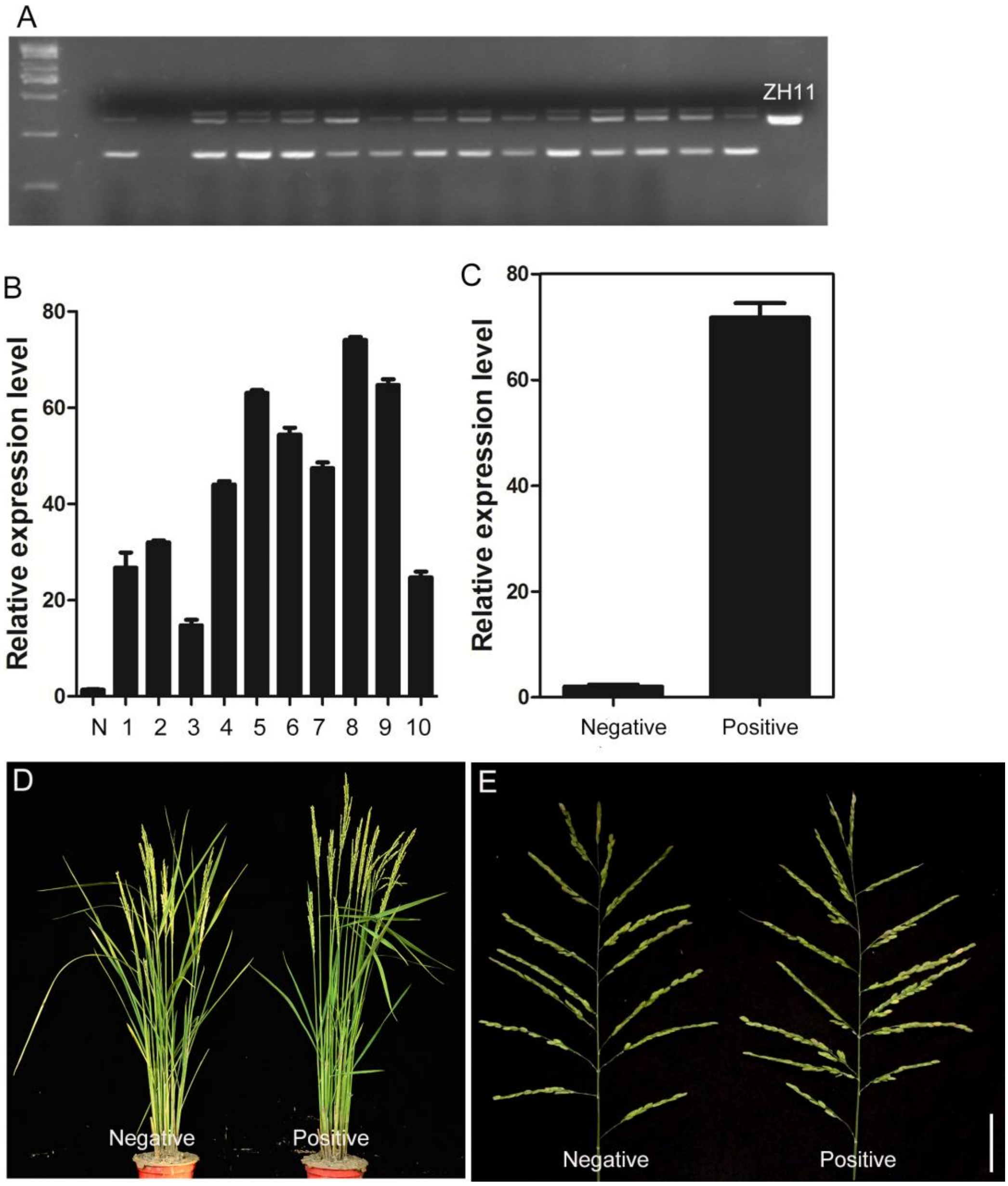

Figure S5. Identification of plants overexpression *VPB1*. (A) Identify the genotype of overexpression plants. (B-C) The qRT-PCR results showed that the VPB1 gene of the positive plants were all overexpressed, 3 biological replicates. (D) Identify the phenotype of overexpression plants. (E) Comparison of panicle type between over-expression positive plants and negative plants. Scale bar, 2 cm.

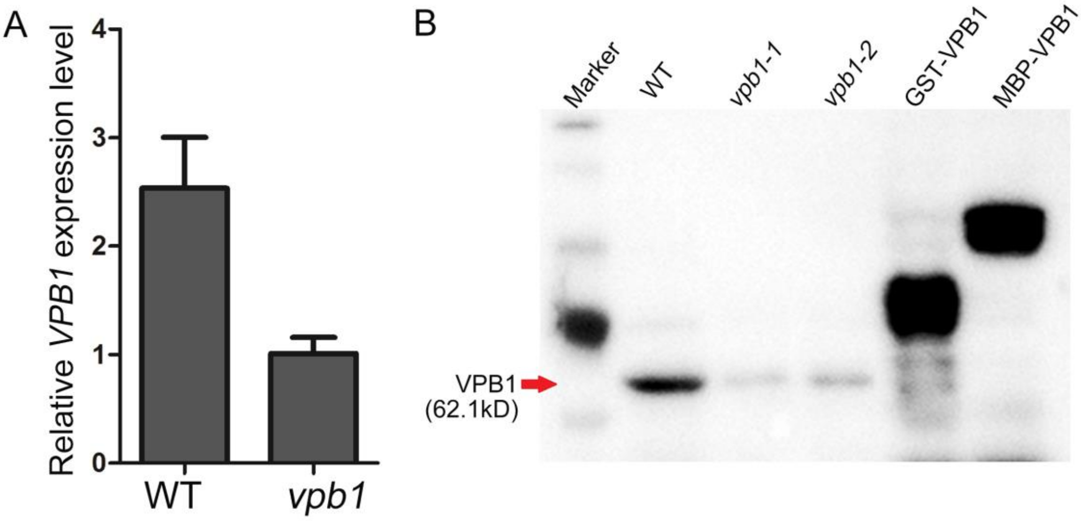

Figure S6. Detection of RNA and protein levels of *VPB1* gene in wild-type and *vpb1* young panicles. (A) Expression level of *VPB1* in young panicles of the *vpb1* mutant and wild-type plants. Data are mean ± SD (n=3 biological replicates). (B) Immunoblots for VPB1 using protein extracts from young panicle of 2-3mm and GST-VPB1, MBP-VPB1. WT: wild type.

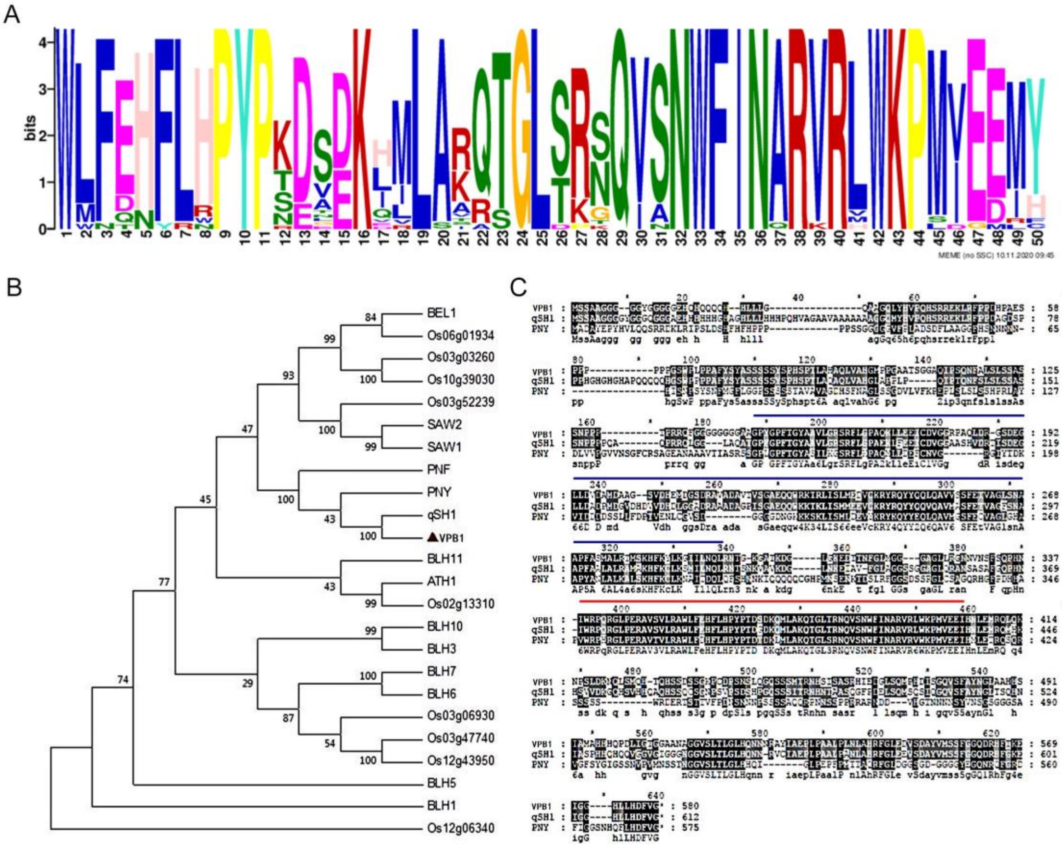

Figure S7. Phylogentic analysis and alignment of the VPB1 amino acid sequence with BLH proteins. (A) The conserved BELL domain was analyzed using the MEME (http://meme-suite.org/tools/meme) v5.0.1 software online. (B) Phylogenetic tree of VPB1 and the functionally identified BEL1-like homeobox(BELL) proteins from rice and Arabidopsis. Phylogenetic analyses were conducted with ClustalX and MEGA6.0, Full-length amino acid residues were used Tree was generated by Neighbor-joining method. The red square indicates VPB1 protein. (C) Alignment of the VPB1 and qSH1

deduced amino acid sequence with Arabidopsis PNY. The shaded letters indicate the identical amino acid residues. Blue line represents SKY BELL domain; red line represent HD domain.

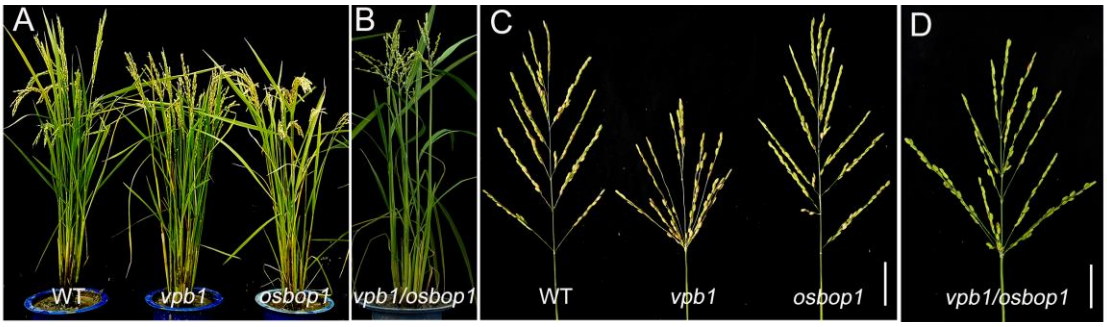

Figure S8. Genetic interactions between *vpb1* and *osbop1* in WT. (A) From left to right is WT, *vpb1*, *osbop1*; (B) vpb1/osbop1 double mutant; (C) the panicle phenotype of WT, *vpb1*, *osbop1* from left to right; (D) the panicle phenotype of *vpb1/osbop1* double mutant. Scale bar, 2 cm.

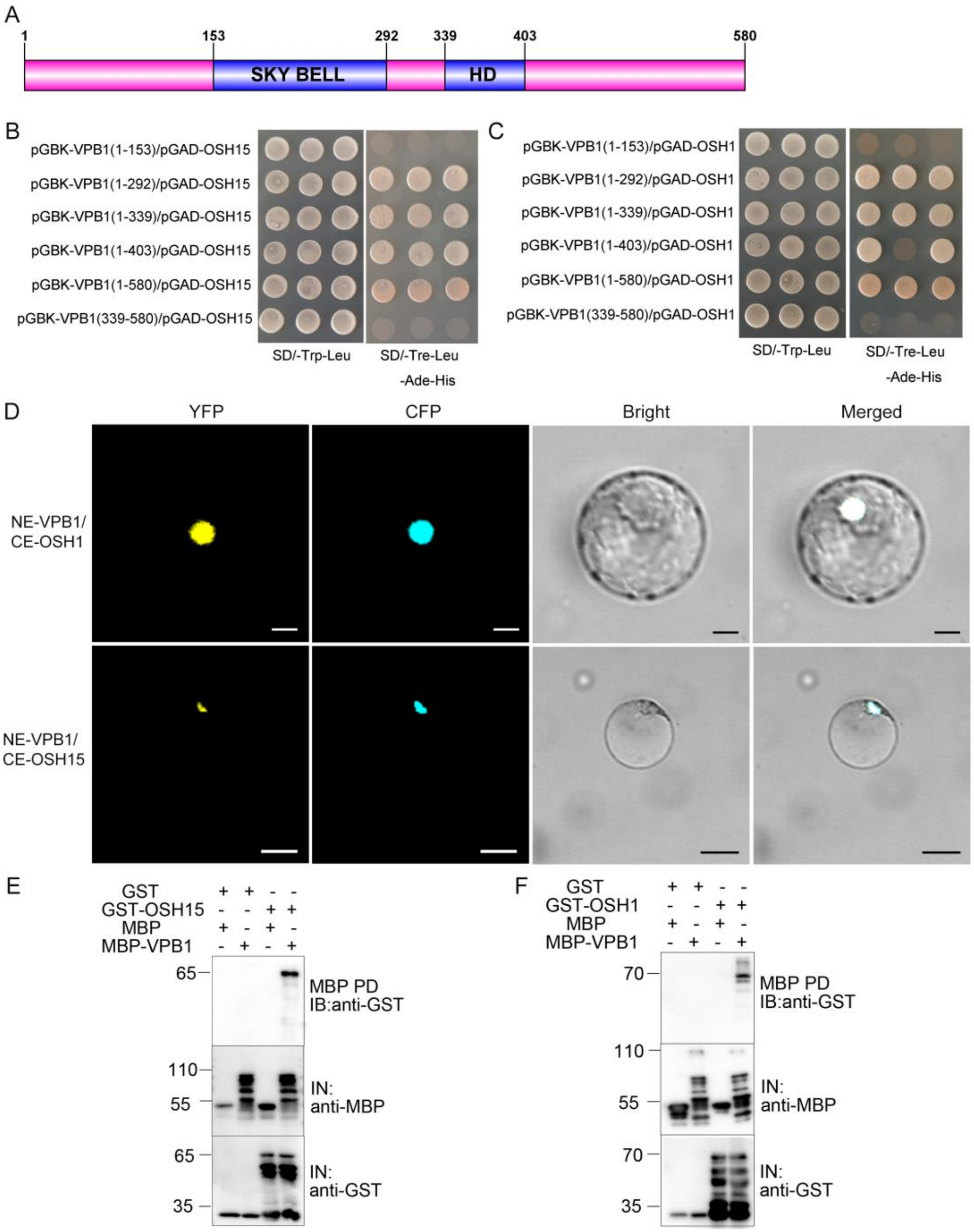

Figure S9. Interaction of VPB1 with OSH1 and OSH15 in vivo and in vitro. (A) Domain structure of VPB1 protein. BELL, BEL-like; SKY, Ser, Lys, and Tyr residues; HD, homeodomain. (B-C) Interaction between VPB1 and OSH1(B) OSH15(C) in the yeast two-hybrid assay. schematic diagram of full-length and a series of truncated VPB1 fusions to DNA binding domain (BD); OSH1 and OSH15 with the DNA activation domain (AD). (D) Bimolecular fluorescence complementation (BiFC) assays of VPB1 interaction with OSH1 and OSH15 in rice protoplasts. YFP fluorescence signal indicates the VPB1 protein localized to the nucleus; CFP fluorescence signal indicates the positive interaction; CFP fluorescence and differential interference contrast (bright) are shown separately and merged. Scale bars, 10 μm, 30 μm. (E-F) In vitro pull-down assay demonstrating the direct interaction between VPB1 and OSH1 (E), OSH15 (F). MBP-VPB1 was pulled down (PD) by GST-OSH1/15 immobilized on MBP beads and analyzed by immunoblotting (IB) using an anti-GST antibody. Each input (IN) lane was immunoblotted using an anti-MBP or anti-GST antibody.

Table S1. Prediction of candidate genes in the fine mapping.

Accession number	Annotation
LOC_Os05g38090	hypothetical protein
LOC_Os05g38100	retrotransposon protein
LOC_Os05g38110	retrotransposon protein
LOC_Os05g38120	homeodomain protein, putative, expressed
LOC_Os05g38130	expressed protein

Table S2. Differentially Expressed Genes between the WT and the *vpb1* Mutant Detected Using RNA-seq.

Table S3. Binding region analysis of the promoter of target genes regulated by VPB1.

Table S4. Primers used in this study.
